# Synthesis and Phytotoxic Activity of New Pyridones Derived from 4-Hydroxy-6-Methylpyridin-2(1*H*)-one

**DOI:** 10.3390/molecules14124973

**Published:** 2009-12-01

**Authors:** Antonio Jacinto Demuner, Vania Maria Moreira Valente, Luiz Cláudio Almeida Barbosa, Akshat H. Rathi, Timothy J. Donohoe, Amber L. Thompson

**Affiliations:** 1 Department of Chemistry, Federal University of Vicosa, Av. P. H. Rolfs, s/n, 36570-000, Vicosa, MG, Brazil; E-Mails: ademuner@ufv.br (A.J.D.); vvalente@ufv.br (V.M.M.V.); 2 Department of Chemistry, University of Oxford, 12 Mansfield Road, Oxford OX1 3TA, UK; E-Mails: akshat.rathi@chem.ox.ac.uk (A.H.R.); timothy.donohoe@chem.ox.ac.uk (T.J.D.); amber.thompson@chem.ox.ac.uk (A.L.T.)

**Keywords:** pyridine, x-ray, phytotoxicity, weeds, herbicides

## Abstract

Commercial dehydroacetic acid was converted into 4-hydroxy-6-methylpyridin-2(1*H*)-one (**3**), which was then condensed with several aliphatic aldehydes to produce seven new title compounds in variable yields (35–92%). Reaction of **3** with α,β-unsaturated aldehydes resulted in the formation of condensed pyran derivatives **4g’** and **4h’**. A mechanism is proposed to explain the formation of such compounds. The effects of all methylpyridin-2(1*H*)-one derivatives on the development of the dicotyledonous species *Ipomoea grandifolia* and *Cucumis sativus* and the monocotyledonous species *Sorghum bicolor* were evaluated. At the dose of 6.7 × 10^-8^ mol a.i./g substrate the compounds showed some phytotoxic selectivity, being more active against the dicotyledonous species. These compounds can be used as lead structures for the development of more active phytotoxic products.

## 1. Introduction

Pyridin-2(1*H*)-ones are known to possess a range of biological activities such as analgesic, antifungal, antimalarial, antiinflammatory, antibacterial, anti-HIV, phytotoxic, antitumoral and antiviral properties [1,2,3,4,5,6,7,8,9]. Functionalized pyridin-2(*1H*)-ones have been used as versatile intermediates in the synthesis of a wide range of nitrogen-containing heterocycles, such as pyridine, quinolizidine, and indolizidine alkaloids [10,11]. Fluridone and sapinopyridione are representative examples of pyridinones that possess phytotoxic activity [5,7]. This class of compounds has been used to formulate lead compounds for the synthesis of new pyridiones [1,3,4,12,13], including the bis(pyridyl)methanes, which present antitumoral activity [8,9]. Recently, we have been utilizing natural products as models for the development of new compounds with phytotoxic activity [14,15,16,17,18,19,20,21,22,23,24]. In continuing our research in this direction, we now report the synthesis of pyridone derivatives **4a-h** and some studies on their phytotoxic activities.

## 2. Results and Discussion

### 2.1. Synthesis of bis-pyridones

**Scheme 1:**
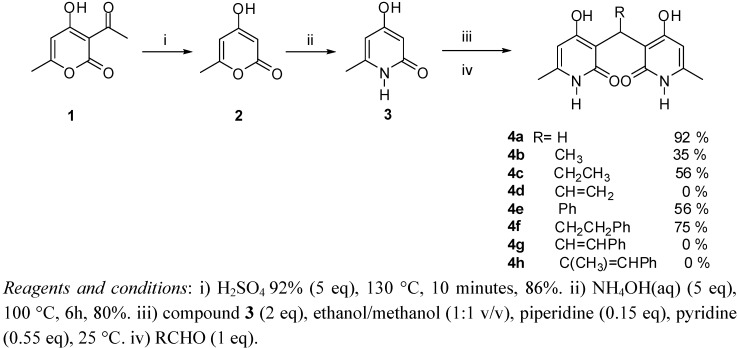
Reagents and conditions for the synthesis of pyridones **4a-h**.

The hydrolysis of dehydroacetic acid **1** [25] with sulfuric acid afforded compound **2** in 86% yield. Compound **2** was then reacted with aqueous ammonium hydroxide to produce the corresponding pyridone **3** in 80% yield. Treatment of **3** under basic conditions in the presence of several aldehydes resulted in the condensed compounds **4a-h** in variable yields (Scheme 1). 

It was found that formaldehyde reacted very quickly and efficiently, resulting in a 92% yield of the condensed bis-pyridone **4a**. In the cases of acetaldehyde and propanaldehyde the reactions were not clean, giving the desired condensation compounds in much lower yields (**4b**, 35%; **4c**, 56%) and several side products were observed by TLC analysis of the crude reaction mixtures. To study the influence on the biological activity of an unsaturated group at the methine carbon, acrolein was reacted with compound **3** under the same conditions described. In this case the required bis-pyridone was not isolated and only polymerized compounds were detect by ESI-MS and NMR of the crude reaction mixture. 

**Figure 1:**
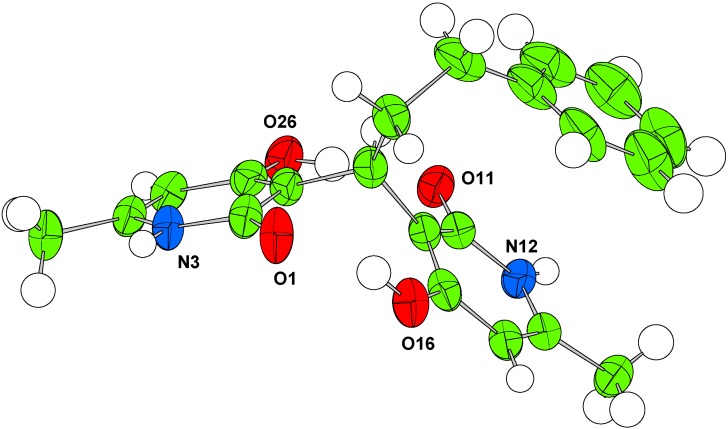
Single crystal structure of compound **4f**. Atomic displacement parameters shown at 50% probability and the disorder are omitted for clarity.

In order to further explore the Structure Activity Relationships (SAR), the preparation of condensed bis-pyridone containing aromatic groups linked to the methine carbon bridge was attempted. Thus, the reaction of benzaldehyde and 2-phenylpropanaldehyde with **3** resulted in the formation of the corresponding bis-pyridones **4e** and **4f** in 56% and 75% yields, respectively. The structures of all condensed products were determined by spectroscopic analysis. In the case of compound **4f**, the structure was also determined by X-ray analysis (Figure 1). 

**Scheme 2:**
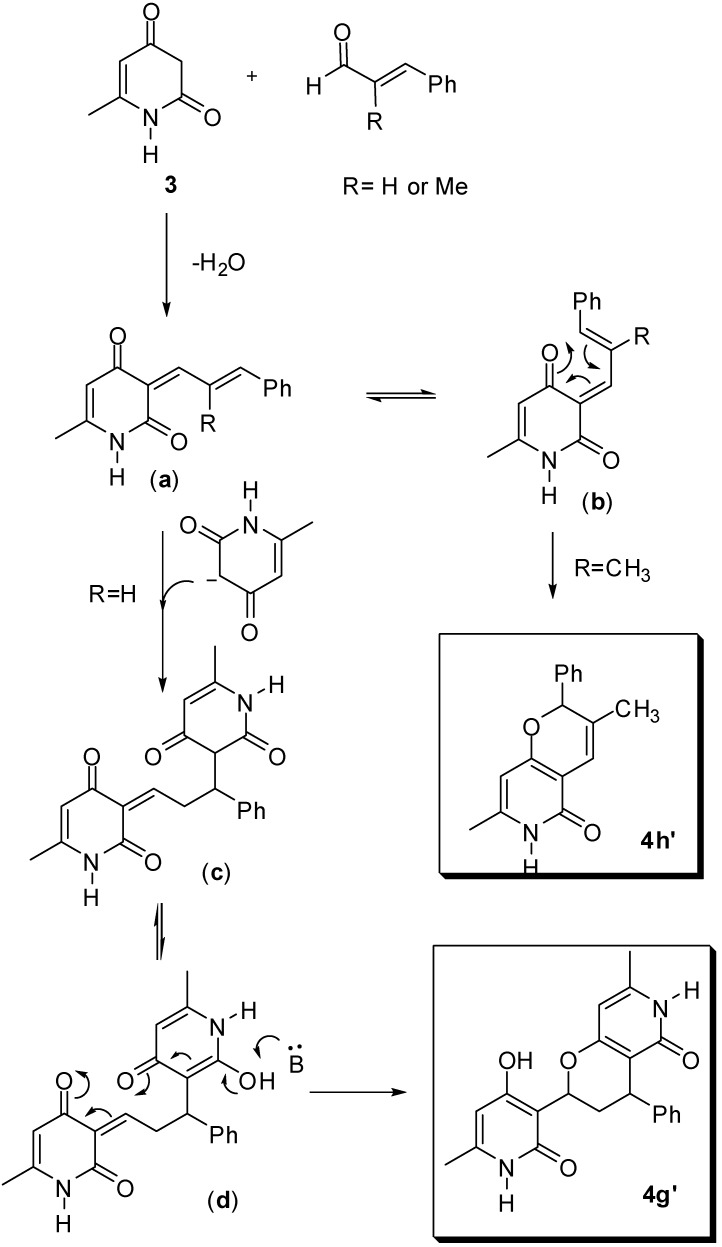
Proposed mechanism for formation of **4g’** and **4h’**.

**Figure 2:**
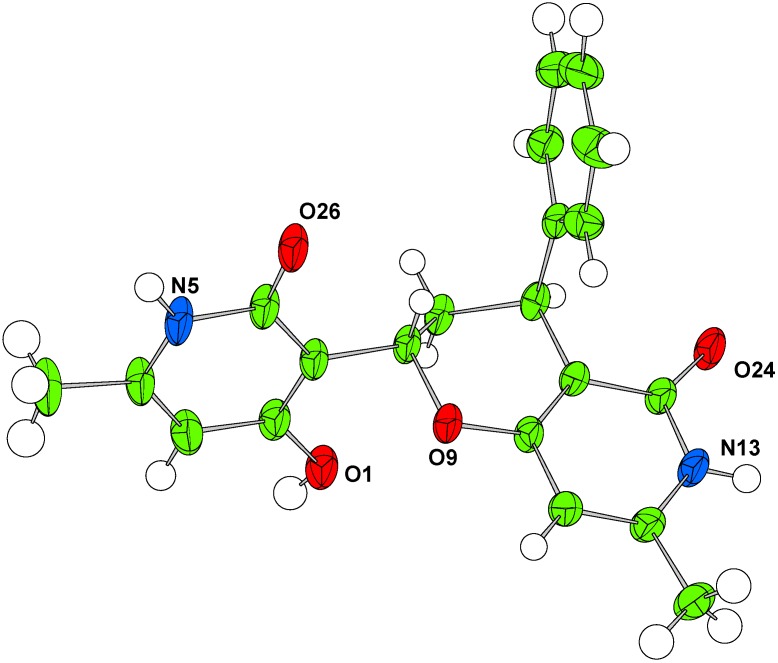
Single crystal structure of compound **4g’**. Atomic displacement parameters shown at 50% probability, one equivalent and the disordered DMSO are omitted for clarity.

**Figure 3:**
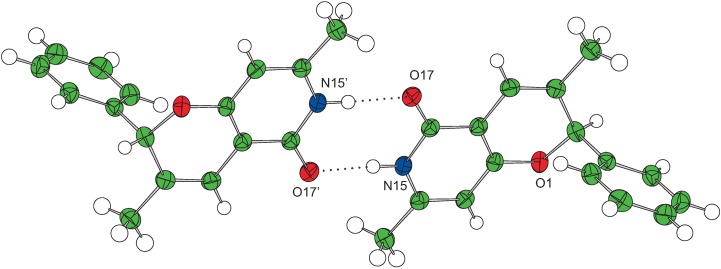
Single crystal structure of **4h’** showing the hydrogen bonding amide dimer. Atomic displacement parameters are shown at 50% probability.

Unexpectedly the reaction of pyridione **3** with cinnamaldehyde and (*E*)-2-methylcinnamaldehyde did not give the desired condensed products. In case of the cinnamaldehyde adduct the HR-MS showed a peak at *m/z* =387.1301, corresponding to the molecular formula C_21_H_20_NaN_2_O_4_. The ^13^C-NMR spectrum showed signals corresponding to two CH_3_, one CH_2_, six CH, two C=O and seven non-hydrogenated carbons. For the (*E*)-2-methylcinnamaldehyde adduct, the product isolated as a white solid with HR-MS showed a peak at *m/z* = 276.0095 corresponding to the molecular formula C_16_H_15_NaNO_2_. The ^13^C-NMR spectrum showed signals corresponding to two CH_3_, six CH, one C=O and five non-hydrogenated carbons. Single crystal diffraction data were collected for both **4g’** and **4h’** yielding the structures shown in Scheme 2, Figure 2, Figure 3. Both compounds were formed as a racemic mixture. Formation of a compound similar to **4h’** has been reported by Lee *et al*. [26], however in none of the published examples a compound with the structure of **4g’** was observed. 

Although no mechanistic investigation was carried out, a pathway is proposed that leads to the formation of **4g’** and **4h’** (Scheme 2). Initially, an aldol condensation of pyridone **3** with cinnamaldehyde results in the intermediate (**a**). Depending on the nature of the R group (H or CH_3_) the product formed in the next step is different. In case of cinnamaldehyde a 1,6-addition of a second molecule of the pyridone resulting in the intermediate (**c**) occurs. This intermediate can be in equilibrium with (**d**), which under basic conditions cyclises to yield **4g’**. In case of (*E*)-2-methylcinnamaldehyde, steric hindrance involving the methyl group and the oxygen favors the formation of (**b**). This intermediate then undergoes a 3,3-sigmatropic rearrangement to give **4h’**. This rearrangement ensures that a second addition is impossible. At this point we should observe that according to the proposed mechanism a concentration of pyridine could have some influence on the results of the reactions, leading to different products, but this is open to further investigation.

### 2.2. Phytotoxicity assay

All the compounds were evaluated against *S. bicolor, C. sativus* and *I. grandifolia,* using sand as a substrate. Although the compounds could have different adsorption properties on sand and this could affect the biological activity, this aspect was beyond the scope of the current work. The effect of compounds **4a-c**, **4e-f, 4g’** and **4h’** on the radicle growth of *Sorghum bicolor* and *Cucumis sativus* was evaluated at 5 × 10^-4^ mol L^-1^ concentration (Table 1). All the compounds caused less than 50% inhibition of both tested plants. The most active compounds were **4g’** and **4h’**, should be causing around 45% inhibition of the root growth of *S. bicolor* and *C. sativus*.

**Table 1:** Effects of compounds **4a-c** and **4e-h** (5 × 10^-4^ mol L^-1^) on the radicle growth of *S. bicolor* and *C. sativus* seedlings after 72 hours.

Compound	*C. sativus* inhibition (%)*	*S. bicolor* inhibition (%)
**4a**	27.6 Ca	27.1 Ba
**4b**	31.2 BCa	23.9 Ba
**4c**	30.2 BCa	26.9 Ba
**4e**	36.5 ABCa	18.9 Bb
**4f**	38.5 ABCa	25.0 Bb
**4g’**	41.0 ABa	44.3 Aa
**4h’**	45.3 Aa	44.3 Aa

* Mean values in the same column with the same capital letters are not significant at P = 0.05% by Tukey’s test; Mean values in the same line with the same small letter are not significant at P = 0.05% by Tukey’s test.

Once all compounds showed some activity on the Petri dish test they were submitted to a 21 day assay in a greenhouse using *S. bicolor*, *C. sativus* and the weed *Ipomoea grandifolia* as test plants (Table 2). 

**Table 2:** Effect of compounds **4a-c**, **e-h** (6.7 × 10^-8^ mol a.i./g substrate) on the biomass production of radicle growth of *S. bicolor* and *C. sativus* seedlings after 21 days.

-	*S. bicolor*		*C. sativus*		*I. grandifolia*	
	aerial part*	roots	aerial part	roots	aerial part	roots
**4a**	22.3 CDc	37.4 ABb	42.6 Bb	65.0 BCa	64.4 Aa	38.6 DEb
**4b**	31.8 BCc	48.0 Ab	63.7 Aa	80.8 Aa	49.8 Bb	45.5 CDb
**4c**	51.6 Ab	44.2 Ab	63.5 Aa	63.9 BCa	68.4 Aa	72.8 Aa
**4e**	18.1 Db	31.7 Bb	34.9 BCa	67.5 BCa	32.8 Ca	32.8 Eb
**4f**	40.2 ABa	38.6 ABb	38.7 BCa	57.6 Ca	45.6 BCa	44.3 CDEb
**4g’**	42.7 ABb	46.8 Ac	38.6 BCb	74.5 ABa	56.8 ABa	58.2 Bb
**4h’**	38.1 Bb	42.8 ABb	27.5 Cc	63.8 BCa	49.3 Ba	55.3 BCa

* Mean values in the same column with the same capital letters are not significant at P = 0.05% by Tukey’s test; Mean values in the same line with the same small letter are not significant at P = 0.05% by Tukey’s test.

In the case of *S. bicolor* compounds **4c**, **4f** and **4g’** caused higher inhibitory effect on the development of the aerial parts (51.6%, 40.2% and 42.7%, respectively). On the other hand, **4b**, **4c**, and **4g’** caused greatest root inhibition development (48.0%, 44.2% and 46.8%, respectively). The results observed for compound **4g’** confirm its higher activity found in the Petri dish test in relation to the other compounds. 

For *C. sativus,* a dicotyledonous species, compounds **4b** and **4c** most severely inhibited the development of the aerial parts (63.7% and 63.5%, respectively). All the other compounds, including the pyrans **4g’** and **4h’**, caused less than 50% inhibition on the aerial parts. The development of the roots of *C. sativus* was most affected by compounds **4b** (80.8% inhibition) and **4g’** (74.5%). All the other compounds were also very active against this dicotyledonous species, causing increases inhibition of the development of their roots compared to the aerial parts. 

Having confirmed the phytotoxicity of the pyridone derivatives against the monocotyledon and dicotyledonous test species, their effect on the development of the important dicotyledonous weed species *I. grandifolia* were also evaluated. For this species, compounds **4a**, **4c** and **4g’** inhibited the development of the aerial part by 64.4%, 68.4% and 56.8%, respectively. Compound **4c** was by far the most effective in inhibiting the roots of *I. grandifolia* 72.8%), followed by compounds **4g’** (58.2% inhibition) and **4h’** (55.3%). All the remaining compounds inhibited the development of the roots of *I. grandifolia* less than 50%. 

The compound **4g’** serendipitously emerged as the most potent inhibitor, showing moderate to high inhibition (38.6% to 74.5%) against all the species tested. Although a clear structure-activity relationship cannot be secured from these data, it is evident that the pyran ring is somehow associated with the activity. It may be concluded that presence of one less pyridone moiety in the molecule does not reduce the phytotoxicity, but rather increases it and that the presence of an aromatic ring at any position (closer or farther from the pyridone ring) encourages higher activity than an alkyl substituent.

## 3. Experimental

### 3.1. General

All the chemicals were purchased from Sigma Aldrich (Milwaukee, WI, USA) and used without purification. The ^1^H- and ^13^C-NMR spectra were recorded on a Varian Mercury 300 instrument (300 MHz and 75 MHz respectively), using deuterated DMSO as a solvent and tetramethylsilane (TMS) as internal standard (δ = 0). IR spectra were obtained using a Perkin Elmer Paragon 1000 FTIR spectrophotometer, using potassium bromide (1% v/v) disks, scanning from 600 to 4000 cm^-1^. Low-resolution mass spectra were recorded on a Fisons Platform instrument under ESI conditions. Chemical ionisation accurate mass spectra were recorded on an Apex III FT-ICR-MS spectrometer with high resolution (resolution = 100,000 FWHM). The lock used for calibration was chloropentafluorobenzene. Values quoted are reported as a ratio of mass to charge in Daltons. Melting points are uncorrected and were obtained from MQAPF-301 melting point apparatus (Microquimica, Brazil). Analytical thin layer chromatography analysis was conducted on aluminum backed precoated silica gel plates. Column chromatography was performed over silica gel (60–230 mesh).

### 3.2. Synthesis

#### 3.2.1. Synthesis of 4-hydroxy-6-methyl-2*H*-pyran-2-one (**2**)

Dehydroacetic acid **1** (1 mmol) and 92% sulfuric acid aqueous solution (5 mmol) were placed in a 25 mL flask and the mixture was heated to 130 °C for 10 minutes. While still warm, the mixture was poured into a beaker containing chopped ice. The resulting precipitate formed was filtered off and washed with cold water, leading to the isolation of 4-hydroxy-6-methylpyran-2-one (**2**) as a white solid in 86% yield. Mp 186.5–187.7 °C; IR: 3,440, 3,097, 2,962, 2,821, 2,733, 2,622, 1,717, 1,657, 1,626, 1,586, 1,540, 1,509, 1,493, 1,344, 1,303, 1,256, 1,191, 1,149, 1,042, 989, 878, 839, 814, 591, 526, and 498 cm^-1^; ^1^H-NMR (DMSO-d_6_) δ: 2.13 (d, 3H, *J* = 0.3 Hz, CH_3_), 5.19 (d, 1H, *J* = 2.1 Hz, H3), 5.93 (m, 1H, H5 ), 11.58 (bs, 1H, OH); ^13^C-NMR (DMSO-d_6_) δ: 20.1 (6-CH_3_), 88.8 (C3), 100.9 (C5), 164.0 (C6), 164.6 (C4), 171.2 (C2); MS, *m/z* (%) 126 (M^+^, 33), 111 (15), 98 (70), 85 (33), 69 (100), 55 (25). 

#### 3.2.2. Synthesis of 4-hydroxy-6-methylpyridin-2(1*H*)-one (**3**)

4-Hydroxy-6-methylpyran-2-one (**2,** 1 mmol) and 28% ammonium hydroxide (5 mmol) were added under stirring to a 25 mL flask. The mixture was heated to 100 °C for 6 h, after which it was cooled in an ice bath and the resultant white solid was filtered off and washed with cold water to provide 4-hydroxy-6-methylpyridin-2-one (**3**) in 80% yield; Mp > 300 °C; IR: 3,445, 3,268, 3,091, 2,894, 2,796, 2,716, 1,653, 1,634, 1,603, 1,550, 1,400, 1,381, 1,352, 1,267, 1,232, 1,175, 1,042, 903, 842, 830, 627, 595, and 535 cm^-1^; ^1^H-NMR (DMSO-d_6_) δ: 2.06 (d, 3H, *J* = 0.3 Hz, 6-CH_3_), 5.32 (d, 1H, *J* = 2.1 Hz, H3), 5.58 (m, 1H, H5), 10.97 (bs, 1H, OH), 10.41 (bs, 1H, NH); ^13^C-NMR (DMSO-d_6_): δ 19.1 (6-CH_3_), 96.4 (C3), 99.0 (C5), 146.6 (C6), 165.53 (C2), 168.3 (C4); MS, *m/z* (%) 125 (M^+^, 77), 96 (22), 84 (100), 69 (17), 68 (17), 55 (16). 

#### 3.2.3. General method of preparation of bis(pyridyl)methanes **4a-h**

To a 100 mL round-bottomed flask, 95% ethanol (20 mL), methanol (20 mL), 4-hydroxy-6-methylpyridin-2-one (**3**, 1.5 mmol), piperidine (15 μL) and pyridine (45 μL) were added. The reaction mixture was kept under stirring for 5 minutes at 25 °C. The corresponding aldehyde (1 mmol) was added and the mixture kept under reflux. On completion of the reaction, as determined by TLC analysis, the volume of solvent was reduced under reduced pressure in a rotatory evaporator and the precipitate formed was filtered and washed with methanol, followed by ethyl ether. This led to the formation of **4a-h** in high purity, without need of any further purification.

*3,3’-Methylenebis(4-hydroxy-6-methylpyridin-2(1H)-one)* (**4a**). Reflux 1.5 h, White solid; yield: 92%; Mp > 300 °C; IR: 3,445, 3,284, 3,059, 3,023, 2,899, 2,644, 1,640, 1,568, 1,442, 1,389, 1,368, 1,267, 1,196, 1,132, 910, 835, 805, 611, and 546 cm^-1^; ^1^H-NMR (DMSO-d_6_) δ: 2.10 (s, 6H, 6-CH_3_ and 6’-CH_3_), 3.41 (s, 2H, H7), 5.86 (s, 2H, H5 and H5’), 11.74 (s, 2H, H1 and H1’), 12.11 (bs, 1H, 4-OH and 4’-OH); ^13^C-NMR (DMSO-d_6_) δ: 18.7 (C7), 18.9 (6-CH_3_ and 6’-CH_3_), 102.1 (C5 and C5’), 109.1 (C3 and C3’), 144.3 (C6 and C6’), 165.6 (C2 and C2’), 167.0 (C4 and C4’); HRMS (ESI TOF-MS): Calcd. for C_13_H_14_N_2_O_4_ 262.0954; found: 262.0951.

*3,3'-(Ethane-1,1-diyl)bis(4-hydroxy-6-methylpyridin-2(1H)-one)* (**4b**). Reflux 90 h, White solid; yield: 35%; Mp > 300 °C; IR: 3,446, 3,264, 2,891, 2,610, 1,644, 1,558, 1,451, 1,390, 1,335, 1,265, 1,198, 1,165, 915, 816, 641, and 553 cm^-1^; ^1^H-NMR (DMSO-d_6_) δ: 1.58 (d, 3H, *J* = 7.5 Hz, H8), 2.09 (s, 6H, 6-CH_3_ and 6’-CH_3_), 4.49 (q, 1H, *J* = 7.5 Hz, H7), 5.81 (s, 2H, H5 and H5’), 11.63 (s, 2H, H1 and H1’); ^13^C-NMR (DMSO-d_6_) δ: 15.3 (C8), 18.7 (6-CH_3_ and 6’-CH_3_), 25.3 (C7), 102.7 (C5 and C5’), 112.9 (C3 and C3’), 144.0 (C6 and C6’), 166.5 (C2 and C2’), 166.7 (C4 and C4’); HRMS (ESI TOF-MS): Calcd. for C_14_H_16_N_2_O_4_ 276.1110; found: 276.1110.

*3,3´-(Propane-1,1-diyl)bis(4-hydroxy-6-methylpyiridin-2(1H)-one)* (**4c**). Reflux 90 h, White solid; yield 56%; Mp > 300 °C; IR: 3,447, 3,057, 2,955, 2,872, 2,622, 1,637, 1,460, 1,393, 1,361, 1,265, 1,252, 1,193, 835, 817, 624, and 557 cm^-1^; ^1^H-NMR (DMSO-d_6_) δ: 0.73 (t, 3H, *J* = 7.2 Hz, CH_3_), 2.03-2.20 (m, 8H, H8, 6-CH_3_ and 6’-CH_3_), 4.18 (t, 1H, *J* = 8.1 Hz, H7), 5.81 (s, 2H, H5 and H5’), 11.60 (s, 2H, H1 and H1’); ^13^C-NMR (DMSO-d_6_) δ: 13.9 (C9), 18.7 (6-CH_3_ and 6’-CH_3_), 21.4 (C8), 33.4 (C7), 102.7 (C5 and C5’), 111.6 (C3 and C3’), 144.2 (C6 and C6’), 166.1 (C2 and C2’), 166.2 (C4 and C4’); HRMS (ESI TOF-MS): Calcd. for C_15_H_18_N_2_O_4_ 290.1267; found: 290.1263.

*3,3'-(Phenylmethylene)bis(4-hydroxy-6-methylpyridin-2(1H)-one)* (**4e**). Reflux 40 h, White solid; yield: 56%; Mp > 300 °C; IR: 3,445, 3,280, 3,062, 2,898, 1,632, 1,453, 1,394, 1,361, 1,284, 1,192, 924, 817, 772, and 583 cm^-1^; ^1^H-NMR (DMSO-d_6_) δ: 2.15 (s, 6H, 6-CH_3_ and 6’-CH_3_), 5.87 (bs, 3H, H-7, H5 and H5’), 6.99 (d, 2H, *J* = 7.5 Hz, H2” and H6”), 7.12 (t, 1H, *J*_1_ = *J*_2_ = 7.5 Hz, H4”), 7.20 (t, 2H, *J* = 7.5 Hz, H3” and H5”), 11.69 (s, 2H, H1 and H1’), 12.31 (bs, 1H, 4-OH and 4’-OH); ^13^C-NMR (75 MHz, DMSO-d_6_) δ: 18.9 (6-CH_3_ and 6’-CH_3_), 34.5 (C7), 102.5 (C5 and C5’), 110.2 (C3 and C3’), 126.0 (C4”), 126.9 (C2” and C6”), 128.5 (C3” and C5”), 139.6 (C1”), 144.8 (C6 and C6’), 167.7 (C2 and C2’), 167.5 (C4 and C4’); HRMS (ESI TOF-MS): Calcd. for C_19_H_18_N_2_O_4_ 338.1267; found: 338.1265.

*3,3'-(3-Phenylpropane-1,1-diyl)bis(4-hydroxy-6-methylpyridin-2(1H)-one)* (**4f**). Reflux 120 h, White solid; yield 75%; Mp 292.5–293.4 °C; IR: 3,446, 3,284, 3,058, 3,042, 3,024, 2,905, 2,622, 1,635, 1,559, 1,455, 1,437, 1,396, 1,257, 1,177, 816, 697, and 552 cm^-1^; ^1^H-NMR (DMSO-d_6_) δ: 2.10 (s, 6H, 6-CH_3_ and 6’-CH_3_), 2.4 (m, 2H, H8), 2.48 (m, 2H, H9), 4.31 (t, 1H, *J* = 7.5 Hz, H7), 5.83 (s, 2H, H5 and H5’), 7.06-7.24 (m, 5H, H2”, H3”, H4”, H5” and H6”), 11.65 (s, 2H, NH-1 and NH-1’), 11.86 (s, 2H, 4-OH and 4’-OH); ^13^C-NMR (DMSO-d_6_) δ: 18.8 (6-CH_3_ and 6’-CH_3_), 30.7 (C8), 30.8 (C7), 35.0 (C9), 102.7 (C5 and C5’), 111.5 (C3 and C3’), 126.3 (C4”), 128.9 (C3” and C5”), 129.0 (C2” and C6”), 137.5 (C1”), 142.5 (C6 and C6’), 167.1 (C2 and C2’), 167.2 (C4 and C4’); HRMS (ESI^+^) C_21_H_22_N_2_NaO_4_ (MNa^+^) required 389.1472, found 389.1479.

*(±)-2-(4-Hydroxy-6-methyl-2-oxo-1,2-dihydropyridin-3-yl)-7-methyl-4-phenyl-3,4-dihydro-2H-pyrano-[3,2-c]pyridin-5(6H)-one* (**4g’**). Reflux 140 h, White solid; yield 57%; Mp 254.8–255.6 °C; IR: 3,418, 3,132, 3,058, 2,932, 1,616, 1,579, 1,455, 1,390, 1,363, 1,321, 1,260, 1,181, 1,155, 1,113, 1,039, 971, 872, 821, 765, 701, 663, and 598 cm^-1^; ^1^H-NMR (DMSO-d_6_) δ: 1.66 (d, 1H, *J* = 13.8 Hz, H8), 2.02 (s, 3H, 6-CH_3_), 2.09 (s, 3H, 6’-CH_3_), 3.10 (dt, 1H, *J* = 13.8, 4.8 Hz, H8’), 4.01 (d, 1H, *J* = 4.8 Hz, H7), 5.12 (dd, 1H, *J* = 12.3, 1.8 Hz, H9), 5.62 (s, 1H, H5), 5.65 (s, 1H, H5’), 7.08 (d, 2H, *J* = 7.2 Hz, H2” and H6”), 7.16 (t, 1H, *J* = 7.2 Hz, H4”), 7.26 (t, 2H, *J* = 7.2 Hz, H3” and H5”), 10.44 (s, 1H, 4’-OH); 11.02 (s, 2H, NH-1 and NH-1’); ^13^C-NMR (DMSO-d_6_) δ: 19.0 (6-CH_3_), 19.1 (6’-CH_3_), 32.5 (C8), 35.4 (C7), 67.2 (C9), 98.5 (C5), 98.7 (C5’), 104.6 (C3), 105.5 (C3’), 126.4 (C4”), 128.6 (C2” and C6”), 128.7 (C3” and C5”), 143.8 (C1”), 146.4 (C6 and C6’), 164.0 (C2), 164.3 (C2’), 164.9 (C4), 166.4 (C4’); HRMS (ESI^+^) C_21_H_20_N_2_NaO_4_ (MNa^+^) required 387.1315, found 387.1301.

*(±)-2-(4-Hydroxy-6-methyl-2-oxo-1,2-dihydropyridin-3-yl)-7-methyl-4-phenyl-3,4-dihydro-2H-pyrano-[3,2-c]pyridin-5(6H)-one* (**4g’**). Reflux 140 h, White solid; yield 57%; Mp 254.8–255.6 °C; IR: 3,418, 3,132, 3,058, 2,932, 1,616, 1,579, 1,455, 1,390, 1,363, 1,321, 1,260, 1,181, 1,155, 1,113, 1,039, 971, 872, 821, 765, 701, 663, and 598 cm^-1^; ^1^H-NMR (DMSO-d_6_) δ: 1.66 (d, 1H, *J* = 13.8 Hz, H8), 2.02 (s, 3H, 6-CH_3_), 2.09 (s, 3H, 6’-CH_3_), 3.10 (dt, 1H, *J* = 13.8, 4.8 Hz, H8’), 4.01 (d, 1H, *J* = 4.8 Hz, H7), 5.12 (dd, 1H, *J* = 12.3, 1.8 Hz, H9), 5.62 (s, 1H, H5), 5.65 (s, 1H, H5’), 7.08 (d, 2H, *J* = 7.2 Hz, H2” and H6”), 7.16 (t, 1H, *J* = 7.2 Hz, H4”), 7.26 (t, 2H, *J* = 7.2 Hz, H3” and H5”), 10.44 (s, 1H, 4’-OH); 11.02 (s, 2H, NH-1 and NH-1’); ^13^C-NMR (DMSO-d_6_) δ: 19.0 (6-CH_3_), 19.1 (6’-CH_3_), 32.5 (C8), 35.4 (C7), 67.2 (C9), 98.5 (C5), 98.7 (C5’), 104.6 (C3), 105.5 (C3’), 126.4 (C4”), 128.6 (C2” and C6”), 128.7 (C3” and C5”), 143.8 (C1”), 146.4 (C6 and C6’), 164.0 (C2), 164.3 (C2’), 164.9 (C4), 166.4 (C4’); HRMS (ESI^+^) C_21_H_20_N_2_NaO_4_ (MNa^+^) required 387.1315, found 387.1301.

*(±)-3,7-Dimethyl-2-phenyl-2H-pyrano[3,2-c]pyridin-5(6H)-one* (**4h'**). Reflux 190 h, White solid; yield 49%; Mp 222.4–223.3 °C; IR: 3,446, 3,058, 2,963, 2,897, 2,746, 1,669, 1,647, 1,630, 1,601, 1,565, 1,485, 1,453, 1,394, 1,263, 1,251, 1,145, 1,035, 951, 907, 869, 803, 770, 763, 696, 663, 649, 627, and 539 cm^-1^; ^1^H-NMR (DMSO-d_6_) δ: 1.61 (s, 3H, 8-CH_3_), 2.04 (s, 3H, 6-CH_3_), 5.59 (s, 1H, H5), 5.77 (s, 1H, H7), 6.40 (s, 1H, H9), 7.33-7.37 (m, 5H, H2’, H3’, H4’, H5’ and H6’), 11.20 (s, 1H, NH-1); ^13^C-NMR (DMSO-d_6_) δ: 19.2 (6-CH_3_), 20.0 (8-CH_3_), 81.0 (C7), 98.0 (C5), 104.4 (C3), 114.5 (C9), 127.0 (C8), 128.1 (C2’ and C6’), 129.4 (C3’ and C5’), 129.5 (C4’), 139.5 (C1’), 145.6 (C6), 160.2 (C2), 161.3 (C4); HRMS (ESI^+^) C_16_H_15_NNaO_2_ (MNa^+^) required 276.0995, found 276.0995.

#### 3.2.4. Single crystal structure determination

The crystal structures of **4f**, **4g’** and **4h’** were determined from single crystal X-ray diffraction data collected at 150 K with an Oxford Cryosystems Cryostream N2 open-flow cooling device [27]. Data were collected using an Enraf-Nonius KappaCCD diffractometer (Mo-Kα radiation (λ = 0.71073 Å) and processed using the DENZO-SMN package [28], including inter-frame scaling (which was carried out using SCALEPACK within DENZO-SMN). The structures were solved using SIR92 [29]. Refinement was carried out using full-matrix least-squares within the CRYSTALS suite [30] on F^2^. A small amount of disorder was present in **4f** (alkyl bridge) and **4g’** (disordered DMSO) which was modeled as detailed in the supplementary information (CIF). Refinement results are included in Table 3 and full crystallographic data have also been deposited with the Cambridge Crystallographic Data Centre, CCDC 755517 – 755519. Copies of these data can be obtained free of charge from The Cambridge Crystallographic Data Centre via www.ccdc.cam.ac.uk/data_request/cif.

**Table 3:** Crystal data and structure refinements for compounds **4f**, **4g’** and **4h**’.

	4f	4g’	4h’
Chemical Formula	C_21_H_22_N_2_O_4_	C_21_H_20_N_2_O_4_·0.421(C_2_H_6_OS)	C_16_H_15_NO_2_
Fw	366.41	396.98	253.11
Crystal system / Space group	*C2/c*	*P*2_1_/*n*	*P* 2_1_/*c*
Crystal colour	Colourless	Colourless	Colourless
Crystal size (mm)	0.22 x 0.16 x 0.12	0.20 x 0.18 x 0.18	0.60 x 0.06 x 0.04
a (Å)	21.8480	20.9365	13.8876
b (Å)	12.1257	9.85390	5.7745
c (Å)	16.7405	21.4160	16.4267
α (°)	90.000	90.000	90.000
β (°)	121.6650	113.2622	104.791
γ (°)	90.000	90.000	90.000
*V* (Å^3^)	3774.71	4059.08	1273.67
**Z**	**8**	**8**	**4**
D_calc_ (g/cm^3^)	1.289	1.300	1.321
*T* (K)	150	150	150
*μ*( Mo Kα) (mm^-1^)	0.09	0.13	0.09
Reflections collected (*R_int_*)	4264 (0.0289)	9268 (0.025)	2923 (0.030)
Data/Restraints/Parameters	2471/0/254	9247/60/552	2130/0/173
*R*_1_ (*I*>2σ(*I*))	0.059	0.084	0.050
*R*_1_ / *wR*_2_ (all data)	0.142	0.123	0.079

### 3.3. Plant growth inhibition assays

In order to evaluate the growth regulatory potential of the synthesized bis(pyridyl)methanes (**4a-h**), two different bioassays were carried out.

#### 3.3.1. Radicle elongation assay on filter paper

The solutions of bis(pyridyl)methanes **4a-h** were prepared by dissolving the compounds in dimethylsufoxide (150 µL). After addition of the surfactant Tween 80 (72 µL), the resulting suspension was transferred to a volumetric flask and diluted with water to 50 mL, to obtain the final concentration 5 × 10^-4^ mol L^-1^. These suspensions were sonicated for 5 min, resulting in complete dissolution of the test compound. Then 4 mL aliquots were used to imbibe two sheets of filter paper (Whatman nº 1) placed in 100 × 15 mm glass Petri dishes. To each dish were added 20 seeds of *Sorghum bicolor* L. Moench (Geneze Company, Paracatu, Minas Gerais State, Brazil) or *Cucumis sativus* L. (purchased from a local market). The plates were incubated at 25 ºC under fluorescent light (8 × 40 W) for 72 h. Radicle length was then measured, and total germination recorded. Seeds were considered to have germinated if a radicle protruded at least 1 mm. Treatments were carried out in a completely randomized design with five replications. The data, expressed as percentage of radical growth inhibition with respect to untreated controls, were analyzed using Tukey’s test at 0.05 probability level. 

#### 3.3.2. Greenhouse trials

Plastic pots (0.13 L) were filled with acid-washed sand, which was saturated with the solution of the test compound (60 mL/450 g of sand, corresponding to 6.7 × 10^-8^ mol a.i./g substrate). Four seeds of *Ipomoea grandifolia*, *C. sativus* or *S. bicolor* were placed in each pot. Seedlings were grown in a greenhouse, and watered as required with tap water or, twice a week, with half-strength Hoagland solution, to maintain the humidity at 13.3% w/w. Twenty-one days after sowing, plants were harvested, and the roots and aerial parts were separated and weighted. Tissues were then dried at 60 °C until there was no further weight loss, and the corresponding dry mass was determined. The percentage of root and aerial part growth inhibition was calculated in relation to the mass of the respective control. Data were expressed and analyzed as previously described [31]. 

## 4. Conclusions

We have described the preparation of a variety of functionalized bis(pyridyl)methanes, starting from dehydroacetic acid. We have demonstrated that the condensation of 4-hydroxypyridone **3** with cinnamaldehydes results in the formation of pyran derivatives. In addition, we have described the effect of the pyridone derivatives prepared on the aerial parts and on the radicle growth of the dicotyledonous species *Ipomoea grandifolia* and *Cucumis sativus* and the monocotyledonous species *Sorghum bicolor*. In general the compounds showed some phytotoxic selectivity, being more active against the dicotyledonous species. It was observed that all tested compounds inhibited the development of the roots of *C. sativus* by more than 50% and three of them inhibit the development of the aerial parts of *I. grandifolia* by more than 50%. Although no concrete structure-activity relationship analysis could be derived due to the limited number of compounds prepared, the phytotoxicity reported for this class of bis(pyridyl)methane substances is an indication of the potential of these substances as lead structures for the development of new compounds with increased activity. Work is currently underway to achieve this goal. 
